# Prediction of the Pharmacokinetic Parameters of Triptolide in Rats Based on Endogenous Molecules in Pre-Dose Baseline Serum

**DOI:** 10.1371/journal.pone.0043389

**Published:** 2012-08-17

**Authors:** Linsheng Liu, Bei Cao, Jiye Aa, Tian Zheng, Jian Shi, Mengjie Li, Xinwen Wang, Chunyan Zhao, Wenjing Xiao, Xiaoyi Yu, Runbin Sun, Rongrong Gu, Jun Zhou, Liang Wu, Gang Hao, Xuanxuan Zhu, Guangji Wang

**Affiliations:** 1 Key Laboratory of Drug Metabolism and Pharmacokinetics, State Key Laboratory of Natural Medicines, China Pharmaceutical University, Nanjing, China; 2 Jiangsu Provincial Hospital of Traditional Chinese Medicine, Nanjing, Jiangsu, China; Rutgers University, United States of America

## Abstract

**Background:**

Individual variances usually affect drug metabolism and disposition, and hence result in either ineffectiveness or toxicity of a drug. In addition to genetic polymorphism, the multiple confounding factors of lifestyles, such as dietary preferences, contribute partially to individual variances. However, the difficulty of quantifying individual diversity greatly challenges the realization of individualized drug therapy. This study aims at quantitative evaluating the association between individual variances and the pharmacokinetics.

**Methodology/Principal Findings:**

Molecules in pre-dose baseline serum were profiled using gas chromatography mass spectrometry to represent the individual variances of the model rats provided with high fat diets (HFD), routine chows and calorie restricted (CR) chows. Triptolide and its metabolites were determined using high performance liquid chromatography mass spectrometry. Metabonomic and pharmacokinetic data revealed that rats treated with the varied diets had distinctly different metabolic patterns and showed differential C_max_ values, AUC and drug metabolism after oral administration of triptolide. Rats with fatty chows had the lowest C_max_ and AUC values and the highest percentage of triptolide metabolic transformation, while rats with CR chows had the highest C_max_ and AUC values and the least percentage of triptolide transformation. Multivariate linear regression revealed that in baseline serum, the concentrations of creatinine and glutamic acid, which is the precursor of GSH, were linearly negatively correlated to C_max_ and AUC values. The glutamic acid and creatinine in baseline serum were suggested as the potential markers to represent individual diversity and as predictors of the disposal and pharmacokinetics of triptolide.

**Conclusions/Significance:**

These results highlight the robust potential of metabonomics in characterizing individual variances and identifying relevant markers that have the potential to facilitate individualized drug therapy.

## Introduction

Individual diversity usually affects the disposition, i.e., the bioavailability, distribution, metabolism and elimination, of a drug *in vivo* and, therefore, accounts for the drug’s level, ineffectiveness and toxicity. Individualized drug therapy is therefore the ultimate goal, which is expected to benefit patients in achieving better therapeutic effects with fewer side effects. Generally, the genetic, lifestyle and environmental factors primarily contribute to individual variances; yet each of them alone cannot fully explain the individual diversity of a drug’s fate *in vivo*. The difficulty of quantifying these multiple confounding factors greatly challenges the realization of individualized drug therapy. In fact, inter-individual variation in response to a drug is strongly inﬂuenced by the patient’s biochemical state [1], which is a consequence of genetic polymorphism, personal genetic variation [2], [3], lifestyles, diet preferences [4], [5], environmental factors [6] or a combination of these factors [7]. The nature of an individual can be manifested in the multi-levels of protein expression, function and activity of enzymes, and fundamental biological metabolism. As the end products of systemic functions and various metabolic pathways, the low-molecular-mass molecules/compounds in biofluids reflect the metabolic patterns and the individual nature of the body. Metabonomic analysis of baseline endogenous molecules provides a possible way to associate individual variances with the fate of a drug *in vivo*. The correlation between basic metabolic patterns and the pharmacokinetic properties of a drug provides a potential approach to understanding the individual diversity of a drug [8], [9]. ‘Pharmaco-metabonomics’ modeling, which was defined as ‘the prediction of the outcome (for example, efficacy or toxicity) of a drug or xenobiotic intervention in an individual based on a mathematical model of pre-intervention metabolite signatures’ [8], is therefore considered as a potentially effective approach for evaluating individual variances for more effective medical treatment.

Biotransformation of the originally active drug into toxic metabolites can lead to side effects and toxicity (e.g., paracetamol is metabolized into *N*-acetyl *p*-quinone imine, NAPQI [10]), but biotransformation of a drug into metabolites of pharmacological activity with less toxicity can reduce side effects or toxicity. For example, triptolide is metabolized into hydroxy-triptolide and shows less toxicity than the parent compound [11]. Various individuals/patients dispose of a drug in different ways, affecting the transformation of the drug and hence the side effects and toxicity. Some pioneering works in pharmaco-metabonomics have suggested the usefulness of metabolic profiling of the fundamental baseline status, i.e., the low-molecular-mass metabolites, in revealing the co-relationship between the disposal patterns of acetaminophen and the metabolic nature of the individuals [9], [12]. Clayton *et al*. [8], [13] successfully predicted the susceptibility to acetaminophen-induced liver injury in rats based on a pre-dose metabolic profile of urine without a previous knowledge of their genotypes. Winnike *et al*. [14] showed that profiles from healthy volunteers obtained shortly after the start of treatment with acetaminophen but prior to alanine aminotransferase elevation, could distinguish toxic responders from non-responders.bThese studies suggested the potential of pharmaco-metabonomics in predicting pharmacokinetic properties of a drug, although the only currently available evidence is for acetaminophen exclusively.

Triptolide is a major bioactive diterpenoid triepoxide isolated from *Tripterygium wilfordii* Hook. F. This compound possesses some distinguishing pharmacological activities for the management of certain intractable diseases, such as lupus erythematosus [15] and diabetic nephropathy [16], and shows anti-fertility [17], anti-inﬂammatory [18], [19], immune-suppressive [19], and antitumor effects [20]. Triptolide also serves as a new molecular probe for studying transcription and potentially as a new type of anticancer agent through the inhibition of XPB ATPase activity [21]. For a long time, triptolide involved traditional Chinese medicine(e.g., Lei Gong Teng Duo Dai Pian, a conventionally available dosage form of triptolide glucosides in China) has been used clinically for the management of arthritis, lupus erythematosus, diabetic nephropathy, tumor, etc. Although for different kinds of diseases the dose varies, the dosage range is strictly defined in order to maximize efficacy while minimize toxicity or side-effects. For an example, the dose of total triptolide glucosides is strictly limited between 1 to 1.5 mg/kg in management of arthritis, and diabetic nephropathy. Animal studies suggested that high amount of triptolide induced evident toxicities, including hepatotoxicity, immunotoxicity, nephrotoxicity, developmental and reproductive toxicities [22], [23], [24], [25]. In addition, studies have shown that metabolism, bioavailability and toxicity of triptolide are heavily dependent on hepatic P450 activities in mice and on sex-related metabolism in rats [26], [27], indicating that metabolism may play an important role in the disposition of triptolide. Among the environmental and lifestyle factors, food preferences primarily contribute to individual diversity within a community and may affect hepatic P450 activities [28], [29] and the disposition of a drug [30], [31], [32]. In this study, to evaluate the possible association between individual diversity and the disposition of triptolide, three groups of rats were provided with different diets to mimic individual diversity. The metabolism and pharmacokinetics of triptolide were evaluated in these rat models, and to represent the individual variances, molecules in the pre-dose baseline serum were profiled with the metabonomic tool of gas chromatography-time of flight mass spectroscopy (GC-TOFMS). Partial least-squares (PLS) modeling was used to screen for potential markers of individual diversity by correlating the molecules in baseline serum and the pharmacokinetic parameters.

## Results

### Liver Histology

Histopathologic inspection of liver slices revealed that high dose of triptolide (1.8 mg/kg) induced distinct karyopyknosis in the control and CR rats, while the toxic effect on HFD rats was less evident. However, low dose of triptolide (0.6 mg/kg) induced perceptible hepatocytic toxicity only in CR rats, Figure S1. Although lipid droplets were obviously accumulated in HFD rats, it appeared negligible hepatic toxicity in the rats fed with HFD chows. The results indicated that rats of the CR chows had more exposure to triptolide than those of control and HFD chows.

### Metabolic Profiling of Molecules in Baseline Serum

**Figure 1:**
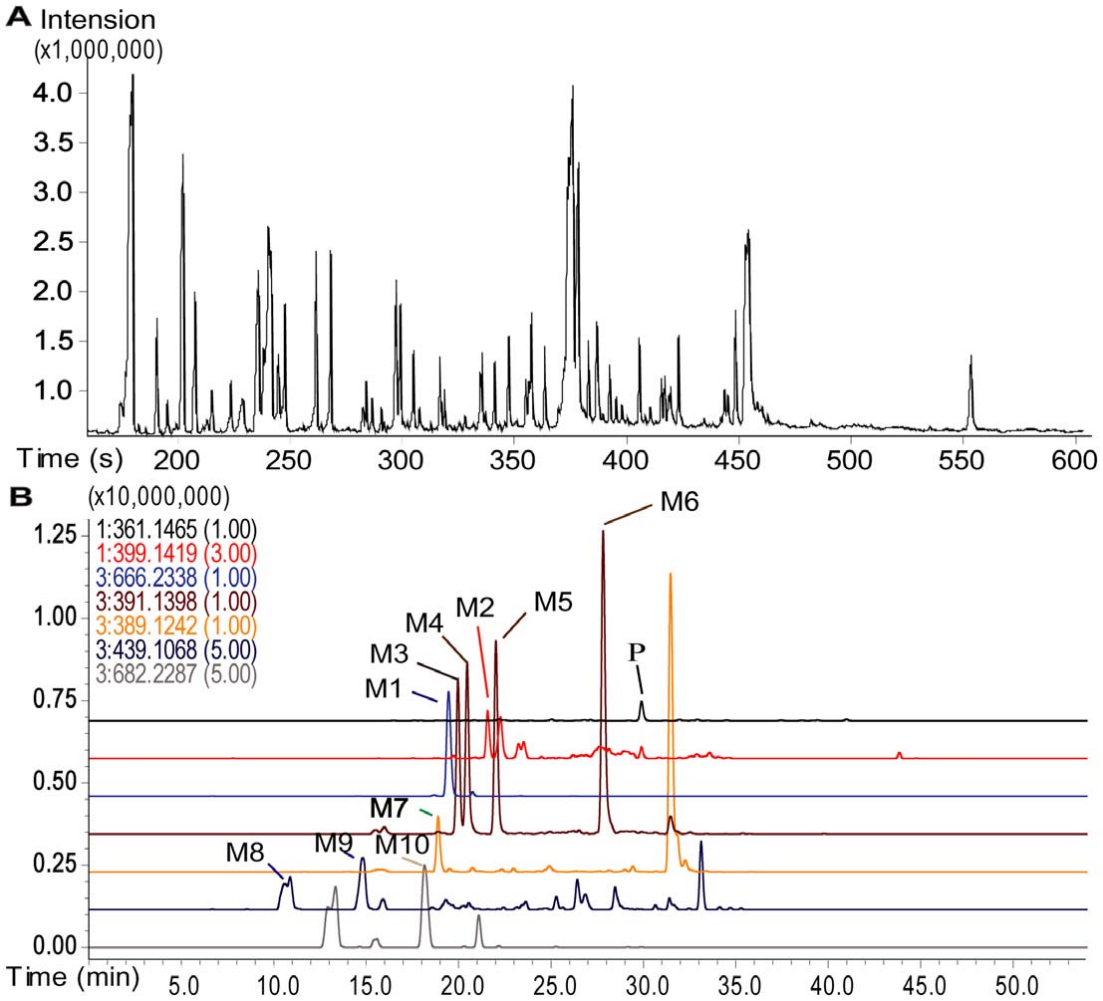
Chromatograms of GC/TOFMS and LC-IT-TOFMS. (A). Typical GC/TOFMS chromatograms of rat blood serum. (B). The LC-IT-TOF/MS-extracted ion chromatograms (EIC) of a bile sample from a rat treated with triptolide. P, parent drug; M1, GSH conjugate of triptolide; M2, mono-hydroxylated triptolide; M3–M6, dihydroxylated triptolide; M7, carboxylated triptolide; M8–M9, triptolide sulfate; M10, mono-hydroxylated triptolide coupled with GSH.

**Figure 2:**
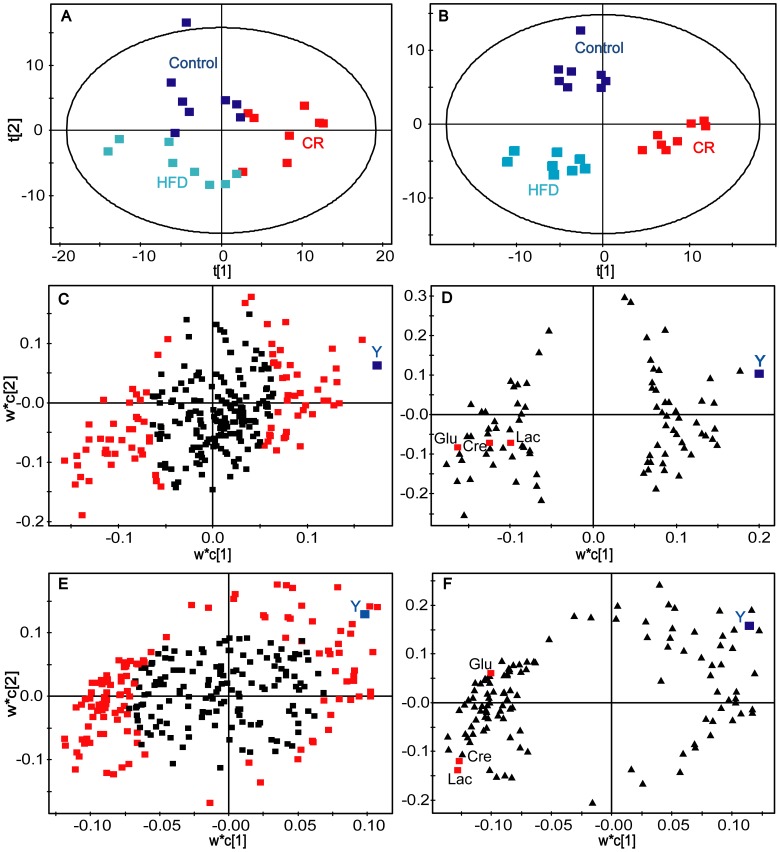
PCA scores plots, PLS-DA score plots and PLS loading plots for predicting the C_max_ of triptolide. (A) PCA scores plots of the three groups (CR, HFD and control). (B) PLS-DA score plots of the three groups (CR, HFD and control). (C) The initial PLS loadings plot based on all the detected peaks/variables. Each point represents a metabolic feature detected in pre-dose serum using GC-MS. Red square, the selected variables of high VIP values were used to build the second PLS model for predicting the C_max_ (high dose). (D) The secondary PLS loadings plot based on the selected peaks with high VIP values. (E) and (F) represent first and second PLS models at low dose, respectively. Glu, glutamic acid; Lac, lactic acid; Cre, creatinine.

In total, the GC/TOFMS analysis of serum detected 267 peaks (Figure 1A), of which 85 were identified, including amino acids, organic acids, carbohydrates, fatty acids, and steroids. The peak area measured in each sample represents the relative intensity of a peak/metabolite monitored in the serum. A peak table was then constructed based on the peak areas with the two vectors (the samples as the observation variables and the peaks as the response variables). An overview of the data in the principal component analysis (PCA) model shows relative clustering of the three groups (Figure 2A), and a partial least squares discriminant analysis (PLS-DA) model clearly shows distant clustering of the three groups (Figure 2B), indicating obvious metabolic differences rendered by the different diets. Identification of the metabolites shows that some molecules in the serum were obviously dependent on the dietary variation, such as glutamic acid, creatinine, lysine, 2-aminobutyric acid, 3-hydroxy-butyric acid, glycine, octadecanoic acid, glutamine, and cholesterol. In detail, 3-hydroxy-butyric acid, glycine, octadecanoic acid, glutamine and cholesterol were more abundant in the CR rats but less concentrated in the HFD rats, while glutamic acid, creatinine, lysine and 2-aminobutyric acid were less abundant in CR rats but more concentrated in the HFD rats. These molecules were therefore suggested as potential markers for indicating individual diversity of the rats.

### Pharmacokinetic Study

**Figure 3:**
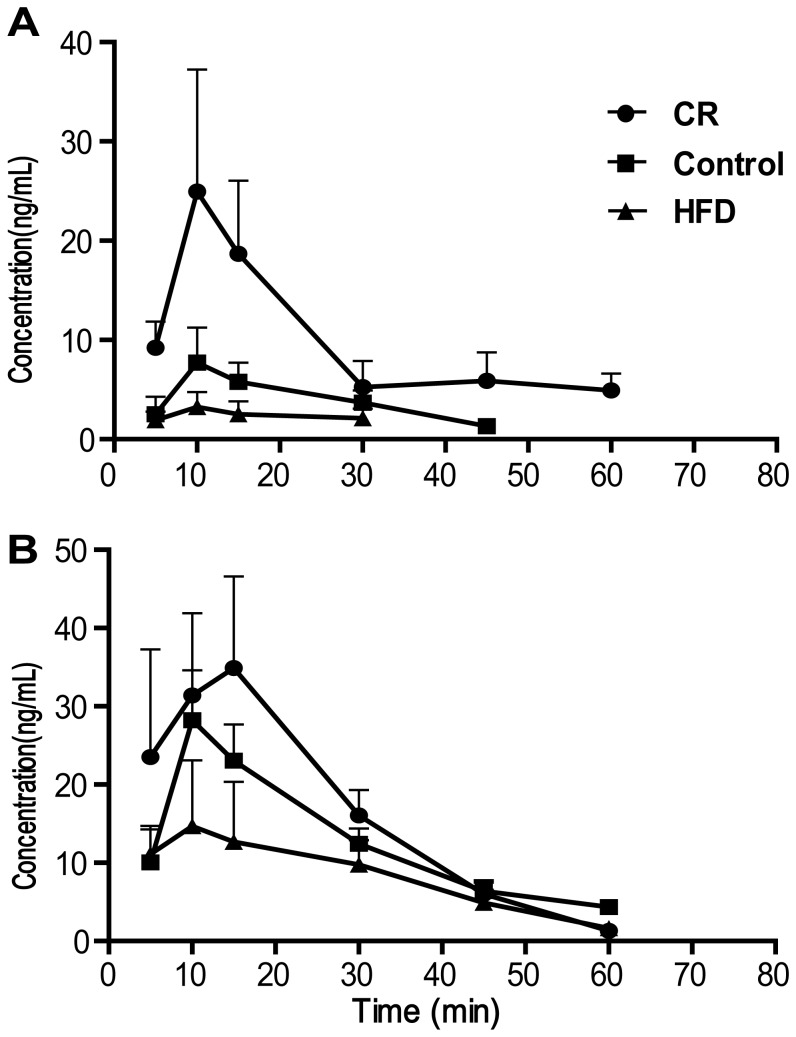
The plasma concentration–time curves of triptolide after oral administration of triptolide. (A) low dose (0.6 mg/kg), (B), high dose (1.8 mg/kg). •, CR diet; ▪, normal diet; ▴, high fat diets. (n = 4, Mean±SD).

To evaluate the pharmacokinetics (PK) of the drug, the plasma concentration of triptolide was measured at various time points after oral administration of the drug (0.6 mg/kg or 1.8 mg/kg). As shown in Figure 3, the plasma concentration–time curves of triptolide reveal a high degree of individual variation in terms of PK. Interestingly, dietary variation greatly affected the PK profiles, where the highest AUC and C_max_ values of triptolide were observed in the CR rats and the lowest AUC and C_max_ values of triptolide were observed in the HFD rats (Table 1), indicating that the PK phenotype of triptolide was dependent on the dietary variation.

**Table 1:** Measured C_max_ and AUC of triptolide in rats plasma.

Parameter	Dose	Control			CR			HFD		
		mean	±	SD	mean	±	SD	mean	±	SD
C_max_ (ng/mL)	Low	7.7	±	3.4	28.9	±	12.5**	3.2	±	1.7#
	High	30.4	±	10.8	37.4	±	11.5	9.8	±	8.2#
AUC(min·ng/mL)	Low	185.7	±	99.1	534.6	±	240.2*	82.1	±	58.3^##^
	High	719.5	±	262.4	964.8	±	312.2	305.3	±	139.8* #

AUC, area under the curve.

* , **Statistically different from the Control, P<0.05 or P<0.01 (t test).

# , ^##^Statistically different from the CR, P<0.05 or P<0.01 (t test).

### Correlation between C_max_ and Baseline Endogenous Molecules

**Figure 4:**
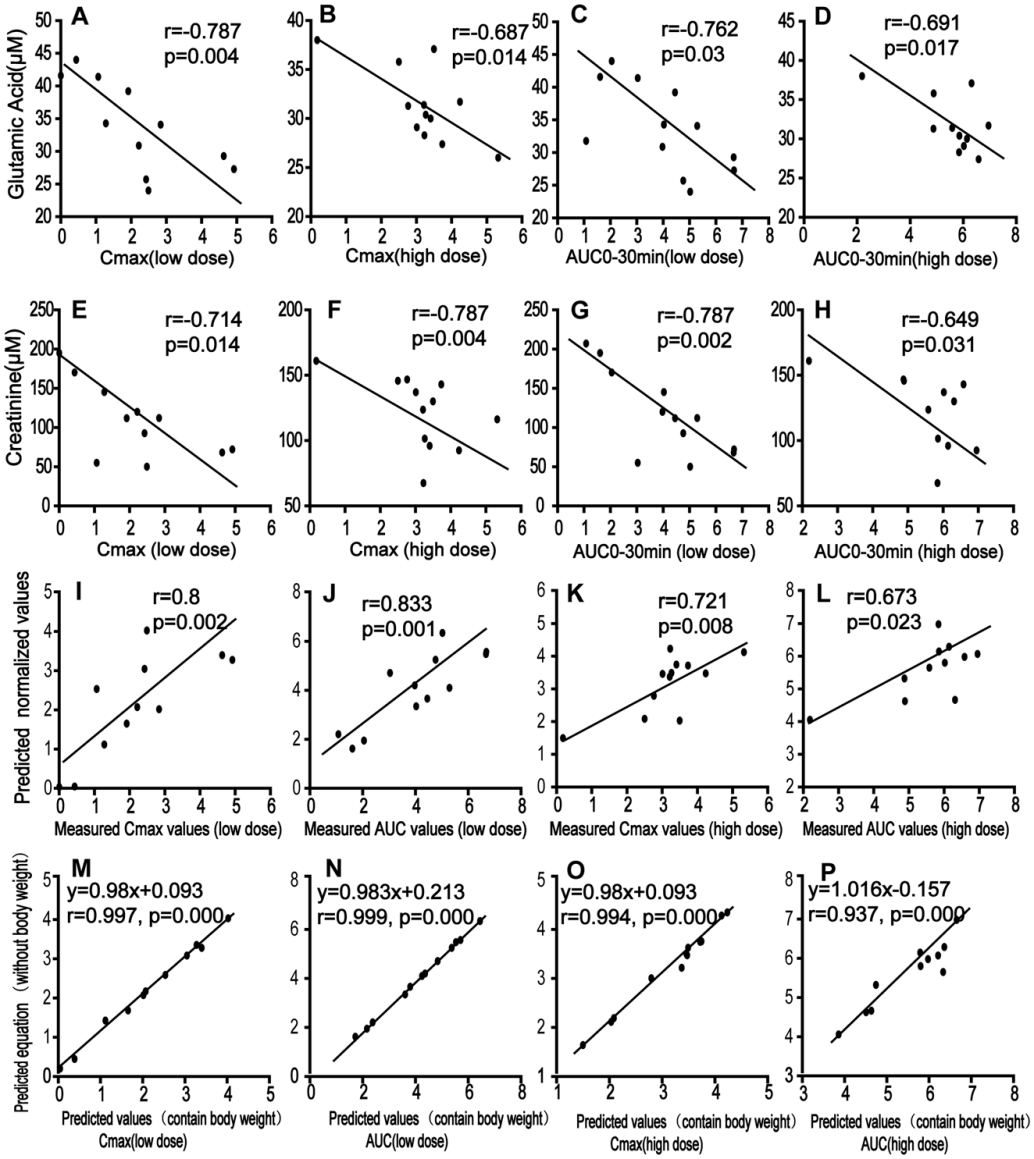
Linear correlation for the prediction of PK parameters of triptolide. (A) C_max_ vs. glutamic acid at low dose; (B) C_max_ vs. glutamic acid at high dose; (C) AUC_0−30 min_ vs. glutamic acid at low dose; (D) AUC_0−30 min_ vs. glutamic acid at high dose; (E) C_max_ vs. creatinine at low dose; (F) C_max_ vs. creatinine at high dose; (G) AUC_0−30 min_ vs. creatinine at low dose; (H) AUC_0−30 min_ vs. creatinine at high dose; (I) measured C_max_ vs. predicted C_max_ values at low dose; (J) measured AUC_0−30 min_ vs. predicted AUC_0−30 min_ values at low dose; (K) measured C_max_ vs. predicted C_max_ values at high dose; (L) measured AUC_0−30 min_ vs. predicted AUC_0−30 min_ values at high dose; (M) Predicted C_max_ values including body weight vs. Predicted values excluding body weight at low dose; (N) Predicted AUC_0−30 min_ values including body weight vs. Predicted values excluding body weight at low dose; (O) Predicted C_max_ values including body weight vs. Predicted values excluding body weight at high dose; (P) Predicted AUC_0−30 min_ values including body weight vs. Predicted values excluding body weight at high dose.

The concentration of triptolide in target organs determines the drug-induced cytotoxicity [11], [33]; C_max_ is therefore an indicator for quantitative evaluation of toxicity. Considering that the ultimate goal of this study was to identify metabolic markers that can characterize individual diversity and predict PK parameters, a two-stage PLS analysis was employed to approach a multivariate statistic model that can predict individualized C_max_ based on the metabonomic data of three dietary groups with two dosages. In the initial PLS analysis, the loadings plot was used to reveal the relevant variables (marked with red squares) that were either positively or negatively correlative to C_max_ (Figure 2C, 2E). The plots/metabolites on the top right corner were positively correlated to the C_max_, while the plots on the lower left quarter were negatively correlated to the C_max_. The variables/molecules correlating to the C_max_ were selected according to the “variable importance to the projection” (VIP) values, which contributed most to the model and the prediction. Finally, the variables/molecules of high VIP values (>1) were selected as basic data (variable data X) in combination with variable Y (the C_max_ values) to build the second PLS model for predicting the C_max_. After cross-validation, the two-component PLS sub-model were built (Figure 2D, 2F) which explained a 60.5% variation in X and predicted a 27.9% variation in Y in the high dose group as well as explained a 36.2% variation in X and predicted a 54.9% variation in Y in the low dose group. Metabolites of high VIP values in the first and second PLS models were selected and correlated to PK parameter (Table S1, such as glutamic acid, creatinine, lactic acid, glyceric acid, valine, uric acid and ornithine. Linear correlation regression revealed that glutamic acid, creatinine, lactic acid, valine, and some unidentified variables correlated well with the C_max_ and that the linearity was independent on the doses. Notably, strong negative correlations for C_max_ to glutamic acid (r = −0.787, p = 0.004 at low dose; r = −0.687, p = 0.014 at high dose [Figure 4A, 4B]) and creatinine (r = −0.714, p = 0.014 at low dose; r = −0.787, p = 0.004 at high dose [Figure 4E, 4F]) were observed.

### Correlation between AUC and Baseline Endogenous Molecules

AUC was calculated and correlated to endogenous molecules in baseline serum. A two-stage PLS model showed that glutamic acid, creatinine, glyceric acid, ornithine and valine had high VIP values in the two PLS model, Table S1. In the PLS model, glutamic acid appeared to be the most promising marker with VIP values of 1.3 and 2.2 at low and high doses, respectively (Figure S2, Table S1). Linear regression showed a strong correlation between glutamic acid and AUC_0−30 min_ (r = −0.762 with p = 0.03 in low dose group, and r = −0.691 with p = 0.017 in high dose group [Figure 4C, 4D]). Creatinine also demonstrated a strong negative correlation to AUC_0−30 min_ (r = −0.787 with p = 0.002 in low dose group, and r = −0.649 with p = 0.031 in high dose group [Figure 4G, 4H]).

### Prediction of C_max_ Based on Levels of Glutamic Acid and Creatinine in Baseline Serum

**Figure 5:**
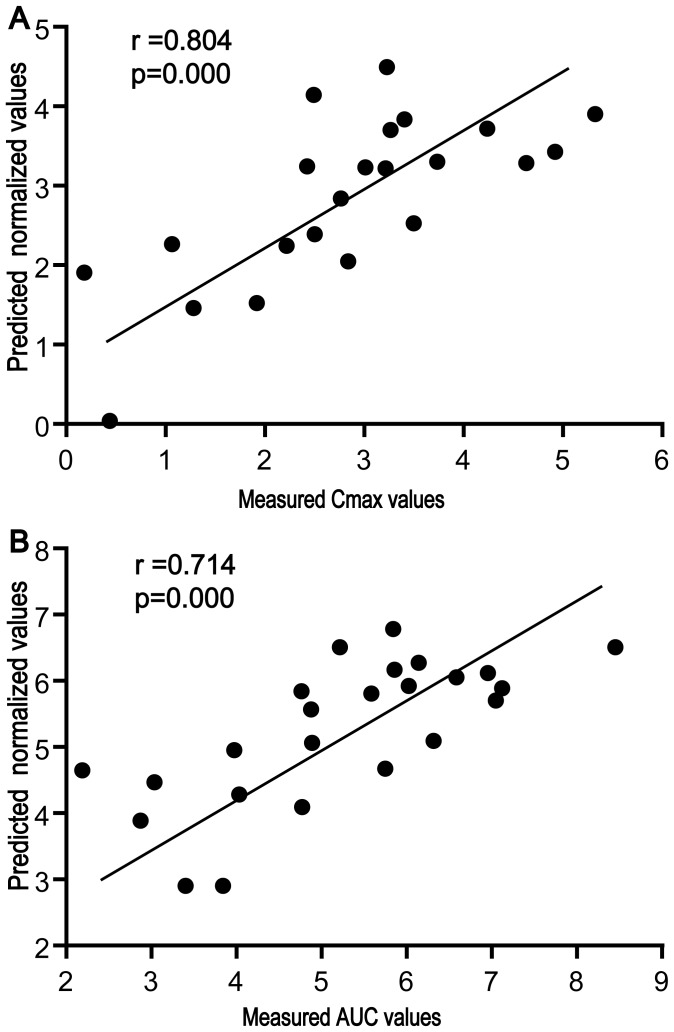
Correlation between the measured and predicted values of PK parameters including both high and low doses of triptolide. (A) measured C_max_ vs. predicted C_max_ values; (B) measured AUC_0−30 min_ vs. predicted AUC_0−30 min_ values.

Based on the measured C_max_ (transformed by the natural logarithm) of triptolide and the concentrations of glutamic acid and creatinine in the baseline serum, linear regression presented two quantitative equations of good coefficients: (1) C_max_pred_ = 6.83−0.077Glu−0.019Cre (C_max_, ng/ml; Glu, µM; Cre, µM), r = 0.800, at the low dose (0.6 mg/kg); and (2) C_max_pred_ = 10.20−0.183Glu−0.010Cre, r = 0.721, at the high dose (1.8 mg/kg). Linear regression between the predicted values and the actual measured values of C_max_ again revealed a good linear correlation (r = 0.800 at the lower dose and r = 0.721 at the higher dose, Figure 4I, 4K), indicating the potential prediction ability based on the baseline data. Considering the possible effect of body weight on the parameters, the linear regression between the C_max_ and the three variables (body weight, glutamic acid, and creatinine) in baseline serum revealed two equations, i.e., C_max_pred_ = 7.878–0.066Glu–0.016Cre–0.007 weight(g), r = 0.803, at the low dose (0.6 mg/kg), and C_max_pred_ = 10.89–0.186Glu–0.007Cre–0.004 weight(g), r = 0.726, at the high dose (1.8 mg/kg). According to the coefficients, the inclusion of body weight as an additional variable resulted in a negligibly improved prediction ability of C_max_ (linear regression coefficients: 0.800 vs. 0.803; 0.721 vs. 0.726), indicating that the variable of body weight contributed little to the prediction ability in addition to the two variables of glutamic acid and creatinine in baseline serum. To approach an integrative equation including data of both high and low doses, a linear regression was applied and the equation was calculated, C_max_pred = _6.921+0.909dose−0.106Glu−0.017Cre (dose, mg/kg, r = 0.804). The predicted values of C_max_ correlated well to the actual measured values (Figure 5A).

### Prediction of AUC Based on Levels of Glutamic Acid and Creatinine in Baseline Serum

The measured AUC (transformed by natural logarithm) of triptolide also showed linear correlation to the concentrations of glutamic acid and creatinine in baseline serum according to the two equations, AUC_0−30 min_pred_ = 9.531−0.087Glu−0.022Cre, r = 0.832, at the low dose (0.6 mg/kg), and AUC_0−30 min_pred_ = 12.436−0.157Glu−0.015Cre, r = 0.673, at the high dose (1.8 mg/kg). The strong correlation between the predicted values and the measured actual values of AUC_0−30 min_ (linear regression coefficients: 0.833 at the low dose, and 0.673 at the high dose [Figure 4J, 4L]) suggests the potential prediction ability based on the baseline data. The inclusion of body weight as an additional variable contributed little to the prediction ability of AUC_0−30 min_, where the equations were expressed as AUC_0−30 min_pred_ = 9.147−0.091Glu−0.023Cre+0.003 weight (g), r = 0.833, at the low dose (0.6 mg/kg) and AUC_0−30 min_pred_ = 14.361−0.149Glu−0.004Cre−0.014 weight (g), r = 0.72, at the high dose (1.8 mg/kg). An integrative equation including data of both high and low doses was calculated, AUC_0−30 min_pred = _9.315+0.797dose−0.114Glu−0.011Cre (dose, mg/kg, r = 0.714). It was shown that the predicted values of AUC_0−30 min_ correlated well to the actual measured values (Figure 5B).

### The Effect of Varied Diets on Metabolites and GSH Conjugates in Bile

Glutamic acid is the primary constituent of glutathione, which plays an important role in detoxification by conjugating with chemicals. To check for evidence of GSH conjugating with triptolide, the metabolites of triptolide in bile were profiled. Based on the accurate masses and the Met ID software supplied in the LC-IT-TOF/MS platform, the quasi molecular ions of [M−H]^−^ and [M+H]^+^ obtained in negative and positive modes, m/z 666.2338 and 668.2500, were identified as a GSH conjugate of triptolide (MW 667.2411); fragmentations confirmed their identification. In detail, the typical loss of 129 Da in the negative mode and 131 Da in the positive mode indicated the presence of a glutamic acid residue that comprises GSH, while m/z 375.13 came from the skeleton of triptolide, where one oxygen was replaced by a sulfur through which triptolide conjugates with GSH (Figure S3). Other metabolites were also identified in bile, including mono-hydroxylated triptolide ([M+Na]^+^ at 399.1419), dihydroxylated triptolide ([M−H]^−^ at 391.1398), carboxylated triptolide ([M−H]^−^ at 389.1242), triptolide sulfate ([M−H]^−^ at 439.1068) and a mono-hydroxylated triptolide coupled with GSH ([M−H]^−^ at 682.2287) (Figure 1B). Consistent with the quantitative results in plasma, the highest amount of the parent drug triptolide was found in the bile from the CR group (0–3 h), while the lowest amount of triptolide was found in the HFD group (Figure S4). Due to the absence of reference standards and the known levels of the metabolites, the relative amounts of the metabolites were semi-quantitatively evaluated by calculating the ratios of peak areas relative to the internal standard. The results showed that in bile, the total molar amount of the metabolites (the GSH conjugate, mono-hydroxylated triptolide-GSH conjugate and di- hydroxylated triptolide, 0–3 h) was higher in the CR group and lower in the HFD group (Table S2). However, in the HFD group, the relative transformation of triptolide (the amount of the metabolites compared to the amount of the parent drug) was much higher than in the CR and control groups (Table S2).

## Discussion

Although pharmacogenetic studies have achieved great success in clarifying individual diversity in PK [2], [3], [34], pharmacogenetics alone cannot fully interpret individual diversity due to the other confounding factors such as environmental and lifestyle factors that also play an important role in affecting the absorption, disposition and elimination of drugs in the body. Among the environmental and lifestyle factors, food preferences contribute to diversity among individuals within a community and may have a great effect on metabolic patterns [35] and the disposition of a drug [30], [31], [32]. Hence, in this study, three groups of rats provided with different diets were chosen to mimic food preferences and to induce individual variances. Of the three diets, different nutritious components and calorie supply primarily contributed to metabolites in serum, while metabolic response of biological systems to dietary change contributed partially, both of which alter metabolic pattern of the rats. It was shown that the three groups had distinctly different metabolic patterns, and a panel of endogenous metabolites in baseline serum characterized the diversity amongst the three groups. Evaluation of baseline metabolomic data and PK parameters of triptolide revealed that some molecules (e.g., glutamic acid and creatinine) in baseline serum correlated well to the C_max_ or AUC value. The two molecules in baseline serum were suggested as the potential markers to represent individual diversity and as predictors of the disposal and PK of triptolide.

To our knowledge, this is the first study to evaluate the predictive capacity of PK parameters (C_max_, AUC) and metabolism of triptolide based on the pre-dose serum molecules. Glutamic acid is well known as the crucial molecule for the synthesis of GSH that plays an important role in the elimination of xenobiotics by conjugating with them [36], [37]. As is known, GSH is involved in anti-oxidative stress and its level reflects the relative response of the rats to various stimuli, herein, high fatty or low calorie diets. It has been reported that chronic caloric restriction elevated GSH levels in rats [38], while GSH levels declined when the rats were induced by high fat diet [39]. Consistently, in this study, more GSH conjugate of triptolide was found in the CR group (relative to the control), while less of them was determined in the HFD group (relative to the control). Unfortunately, the contribution of metabolism to PK parameters remains elusive because the authentic metabolites are not available and the quantitative measurement of various metabolites of triptolide in serum and bile is impossible. However, the reverse correlation between glutamic acid, creatinine and triptolide highlights the potential role of baseline glutamic acid and creatinine in predicting individualized levels of triptolide, the possible side effects and the therapeutic outcomes. In addition, the two suggested markers, glutamic acid and creatinine, are among the most commonly available endogenous metabolites that can be quantitatively determined in most biological samples, irrespective of the origin species (animal or human). This advantage greatly facilitates a translational research from model animals to humans.

Despite the potential dispute of these two molecules as PK markers or as surrogate endpoint markers of individual variance, their significance is obvious. Individualized drug therapy has been proposed for a long time, yet few regimens have been amended or practiced based on environmental or lifestyle factors, largely because the appropriate tools for quantitative measurement of the individual variances are absent. The absence of baseline biomarkers that can predict a drug’s fate (absorption, distribution, metabolism and elimination) within the body greatly hinders our effort to realize an individualized regimen design and to further improve safety and efficacy of an effective pharmacotherapy. Dissimilar to genomics and proteomics, metabonomics permits the quantitative measurement of endpoint metabolites (endogenous low–molecular-weight molecules) that represent the ultimate downstream response of biological systems to genetic or environmental change. A change in metabolic patterns does not only provide an insight on the functional changes that occur due to any given stimuli, both genetic and environmental, but also reflect the underlying biology of the individual [40]. The identified metabolic markers that reflect the nature of an individual in a quantitative way are of crucial significance for the quantitative prediction of PK parameters. We believe that the suggested markers in model rats have the potential to bridge preclinical and clinical usage of triptolide for evaluating PK properties, although further translational research is required.

It is conceivable that the differential C_max_ level of triptolide in the three groups is associated with the body weight of the rats. Indeed, the rats in the HFD group possessed a higher average body weight than those of the normal control group, while the rats in the CR group had the lowest body weight (Table 2). In other words, the body weights of the rats were negatively correlated to C_max_ and AUC values of triptolide. However, the linear regression revealed the poor correlation-ship between PK parameters and body weight alone at higher dose of triptolide (1.8 mg/kg) for both C_max_ and AUC, although the linearity is not bad at lower dose (0.6 mg/kg), Table S3. Further linear regression analysis showed that body weight did not significantly contribute to the variation of the PK parameters of C_max_ and AUC, because inclusion of body weight as an additional variable did not improve the prediction ability for C_max_ and AUC at either the high dose or the low dose, according to their linear coefficients (Figure 4M–4P). On the other hand, levels of glutamic acid or creatinine correlated to body weight to some extent (Figure S5), while the normalized levels of glutamic acid and creatinine (vs body weight) were not well correlated to C_max_ and AUC, especially at higher dose(1.8 mg/kg), Table S4. These results suggest that these two metabolic markers (glutamic acid and creatinine) reflect individual variation well and may have included the contribution of body weight to the prediction ability of PK parameters.

**Table 2:** The body weight of rats fed with different diets for two weeks.

	Control			CR			HFD		
	Mean ±SD			Mean ±SD			Mean ±SD		
Low dose	258.5	±	8.5	222.0	±	15.0**	300.8	±	14.5**
High dose	262.8	±	2.5	219.3	±	11.3**	293.3	±	15.5**

** Statistically different from the control, P<0.01 (t test).

## Materials and Methods

Triptolide was purchased from Suzhou Bochetown Medical Technology Company, Ltd (Suzhou, China). The stable isotope-labeled [^13^C_2_]-myristic acid (Cambridge Isotope Laboratories, Andover, MA, USA) was added to the serum as the internal standard (IS). The alkane series (C_8_–C_40_, Fluka, Buchs, Switzerland) was analyzed as standards to calculate retention index. N-methyl-N-trimethylsilyl-trifluoroacetamide (MSTFA) plus 1% trimethylchlorosilane were provided by Pierce Chemical (Rockford). Methoxyamine was purchased from Fluka (Switzerland). Prednisolone acetate and warfarin were provided by the National Institute for Control of Pharmaceuticals and Biological Products (Beijing, China). Methanol and acetonitrile were provided by Fisher Scientific (USA).

### Animal Experiments and Sample Collection

Twenty-four male Sprague Dawley (SD) rats (weight 180–200 g) were purchased from Slac Laboratory Animal (Shanghai, China). To mimic different lifestyle diets, a two-week dietary intervention study was undertaken: fatty, calorie restricted (CR) and routine diets were supplied with a 12 h light-dark cycle for 2 weeks and water *ad libitum*. Rats were randomly assigned to one of three different experimental groups with 8 rats per group. Group A was provided with the high fat diet (HFD) (2% cholesterol, 10% pork fat, 10% custard powder, 0.5% cholate and 77.5% basal diet), group B with the 40% calorie restricted (CR) diet and group C with the conventional diet as control (China Experimental Animal Food Standard, GB 14924.2–2001 and GB 14924.3–2001, containing 190 g/kg crude protein, 60 g/kg crude fat, 50 g/kg crude fiber, 16 g/kg calcium, and 12 g/kg phosphonium). After an overnight fasting, triptolide was administered intragastrically at a low or a high dose (0.60 or 1.80 mg/kg, respectively, n = 4), and 250 µL of blood was collected at 0, 5, 10, 15, 30, 45, 60, 90, 120, and 180 min. An aliquot of blood was put into a gel and a heparinized tube to prepare serum and plasma, respectively. Plasma (100 µL) was isolated and stored at −70°C for LC/MS analysis of triptolide, and a 30 µL aliquot of pre-dose serum was prepared for GC/TOFMS analysis of the endogenous molecules. The animal experiments were conducted with the approval of the Animal Ethics Committee of China Pharmaceutical University. The use and care of experimental animals complied with all regulations in the Use of Laboratory Animals, as adopted and promulgated by the United States National Institutes of Health.

Bile samples for blanks were collected from model rats fed with HFD, routine diet, and CR. Bile samples were also collected after the rats were intragastrically administered a single dose of triptolide (1.8 mg/kg) for 12 hours. Both the blank bile and bile samples (after treatment) were precipitated and extracted with methanol (bile: methanol, 1∶5), and the supernatants were analyzed in the LC-IT-TOFMS (Shimadzu, Kyoto, Japan) for identifying the metabolites of triptolide and in the LC-MS (Shimadzu, Kyoto, Japan) for quantitative analysis of triptolide and its metabolites.

### Liver Histopathology

After feeding with different diets for 2 weeks, the rats were intragastrically administered triptolide (0.60 mg/kg and 1.8 mg/kg) and then euthanized and sacrificed 24 h later. The livers were then harvested for histological inspection as previously described [25].

### GC/TOFMS Analysis, Data Acquisition and the Identification of Metabolites

Serum samples (50 µL) were prepared, derivatized, and measured as described previously [41]. For chromatographic separation, the derivatized sample (0.5 µL) was injected into a 10 m×0.18 mm ID fused-silica capillary column chemically bonded with 0.18 µm DB-5MS stationary phase (J&W Scientific) in an Agilent 6890 GC system, and the analytes in the eluent were profiled in a Pegasus III TOFMS (Leco Corp., St. Joseph, MI, USA) as described previously [41]. The acquired GC/TOFMS raw data were deconvoluted using the ChromaTOF 3.25 software [42]. The retention index of each peak was calculated by comparing its retention time against those of the standard alkane series C_8_–C_40_. Each metabolite was identified by comparing its mass spectrum and retention index with those of authentic reference standards and those available in the National Institute of Standards and Technology (NIST) library 2.0 (2008), the Wiley 9 library (Wiley_VCH Verlag GmbH & Co.KGaA, Weinheim, Germany), and in-house libraries established by the Umeå Plant Science Center, Umeå University (Sweden), and by the key lab of drug metabolism and PK at China Pharmaceutical University.

### Quantitative Analysis of Triptolide in Plasma

The concentration of triptolide in plasma samples were determined in a Shimadzu 2010A liquid chromatographic/mass spectrometry system (LC/MS, Kyoto, Japan) with an atmosphere pressure chemical ionization (APCI) interface [43]. The quant masses, [M−H]^−^ at m/z 359.15 and 341.3 for both triptolide and the IS (prednisolone acetate), were monitored. The concentrations of triptolide were calculated by fitting the peak area ratios of the analyte to the IS into the calibration curve ranging from 1 to 500 ng/mL. A concentration-time curve was finally graphed, and the area under the curve (AUC) was calculated.

### Quantitative Measurement of Glutamic Acid and Creatinine

The concentrations of glutamic acid and creatinine in serum were determined and calculated by comparing their relative abundances in GC/TOFMS responses in serum with those of standard solutions of glutamic acid and creatinine. Peaks of glutamic acid and creatinine were quantified at the quant masses of m/z 246.1 and 429.3, respectively, and the internal standard, [^13^C_2_]-myristic acid, was quantified at m/z 287.2. The stock solutions of the glutamic acid and creatinine were prepared at 10 mM in water, from which a series of standard working solutions (5, 20, 50,100, 200, and 500 µM) were prepared by diluting both stock solutions with methanol. In each of the diluted standard solutions, 20 µL was removed and added into GC vials. After drying in a speed-vacuum, derivatization, and GC/TOFMS analysis, standard curves were prepared for calculating the concentrations of glutamic acid and creatinine in serum.

### LC-IT-TOF/MS Analysis and the Identification of Metabolites of Triptolide

Liquid chromatography was conducted in a Shimadzu HPLC system (Kyoto, Japan). Chromatographic separation was achieved on a Zorbax SB-C18 3.5 µm 150 mm×2.1 mm ID column (Agilent, USA) at 35°C with a mobile phase rate at 0.2 mL/min. The mobile phase was comprised of solvent A (pure water containing 0.04% HCOOH) and solvent B (methanol). A gradient elution was programmed: 10% solvent B constantly for 0–5 min, a linear gradient of 10–70% solvent B from 5–40 min, which was then maintained for 5 min. Finally, the mobile phase was returned to an initial 10% solvent B within 3 min and maintained for 4 min.

Multiple MS*^n^* analyses were conducted on a Shimadzu IT-TOF-MS equipped with an electrospray ionization (ESI) source, and the optimized operating conditions were as follows. A positive mode with electrospray voltage of 4.5 kV and a negative mode of −3.5 kV were monitored simultaneously. The curve dissolution line (CDL) and the heat block temperature were set at 200°C, with nebulizer gas (N_2_) flows at 1.5 L/min, and cooling gas (Ar) flows at 95 mL/min. The pressure in the ion trap was 1.7×10^−2^ Pa, with an ion accumulated time of 200 ms. The collision energy was set at 15% both for MS^2^ and MS^3^; for MS^1^, *m*/*z* 200–700 were scanned, while *m*/*z* 100–700 were scanned for MS^2^ and MS^3^.

Metabolite identification was carried out using the Met ID solution 1.0 based on the accurate masses of molecular ions and their fragmentations. Shimadzu’s Composition Formula Predictor software was used to calculate the chemical formula for confirming the identification of metabolites of triptolide.

### Measurement of Triptolide and its Metabolites in Rat Bile

Triptolide and its metabolites in rat bile were analyzed on a Shimadzu 2010A liquid chromatographic system equipped with an ESI source. The quantitative measurement of triptolide and its metabolites was performed using LC-IT-TOFMS. The CDL and heat block temperature were set at 250°C and 200°C, respectively, with the detector voltage of 1.6 kV. Liquid nitrogen was used as the nebulizer gas, and the curtain gas source was set at 1.5 and 2.0 L/min. [M+H]^+^ at m/z 361.15, 668.25, 684.20, 413.10, and 415.20 were monitored for triptolide, GSH conjugate, mono-hydroxylated triptolide-GSH conjugate, carboxylated triptolide, and dihydroxylated triptolide, respectively, and the IS of warfarin was detected for [M+Na]^+^ at m/z 331.15. Peak area ratios of the triptolide to IS were calculated by fitting the data into the calibration curve.

### Multivariate Statistical Analysis

For each of the samples, the detected peaks in GC/TOFMS were identified and their peak areas were obtained as previously described [42], [44]. Consequently, the acquired metabonomic data was a data matrix constructed by the peak areas normalized against the internal standard, with two vectors: sample names as observations in the first column, and retention times/peaks as the response variables in the first row. Multivariate statistical analysis (MVSA) was carried out based on the dataset using SIMCA-P 11 software (Umetrics, Umeå, Sweden) as published [45].

As a mathematic model, every sample represents a plot in a N-dimensional space where N stood for the number of variables. Here, principal component analysis (PCA) and partial least squares projection to latent structures & discriminant analysis (PLS-DA) were employed to process the metabonomic data. PCA involves a mathematical procedure that transforms a number of detected variables into a smaller number of ‘dummy’ variables called principal components (PCs), i.e., by projecting the plots and reducing to a few principal components that described the maximum variation of different groups or samples. Thus, the comparative analysis of the data was facilitated by reducing the dimensionality of the data set while retaining the similarity or diversity of the samples as much as possible. The result of PCA displayed as score plots to represent the scatter of samples, which cluster closely to indicate similar metabonomic composition and stand far away to indicate compositionally different metabonome. The purpose of PLS-DA was to calculate models differentiating groups or classes. For PLS-DA modeling, samples from the one group were classified into the same one, respectively, so that all samples were divided into different groups as the qualitative ‘dummy’ variable, Y. Cross-validation [46] with seven cross-validation groups was used throughout to determine the number of principal components (PC), and a permutation test was performed with an iteration of 100. The goodness of fit for a model is evaluated using 3 quantitative parameters; i.e., R^2^X is the explained variation in X, R^2^Y is the explained variation in Y, and Q^2^Y is the predicted variation in Y. The parameters of model were carefully checked to avoid over-fitting of the model.

### Selection of Baseline Molecules Correlating to PK Parameters

In addition, partial least-squares projection to latent structures (PLS) was employed to correlate metabonomic data and PK parameters, i.e., metabolites peak areas from pre-dose serum as predictor variables (X) and the C_max_ or area under the curve (AUC_0-t_, logarized) of triptolide as the response variables (Y). The relevant markers of PK were identified based on the four criteria followed. i) the metabolites must be authentically identified to facilitate further studies; ii) the metabolites had high VIP values (VIP>1) in the PLS models with metabolites in baseline serum as predictor variables (X) and the PK parameters (C_max_ and AUC) of triptolide as the response variables (Y) [9]; iii) The discriminatory metabolites characterized metabolic patterns of the three groups conferred by varied diets, and the relative standard deviations of them were not more than 30% within a group; iv) the metabolites had high coefficients (>0.6, Pearson regression) when they are correlated to C_max_ and AUC.

### Statistical Analysis

The results are shown as the mean±SD, and the Pearson correlation was calculated by SPSS (version 16.0). Statistical analysis between groups was performed using a One-way ANOVA embedded in SPSS (version 16.0) with a significant level of 0.05 or 0.01.

## Supporting Information

Figure S1 **Histopathological inspection of the livers (HE staining) of rats with or without triptolide(0.6 mg/kg).** A: Control+Non-treated; B: Control+ treated; C: CR+Non-treated; D: CR+ treated; E: HFD+Non-treated; F: HFD+ treated.
(TIF)

Figure S2 **PLS loading plots for predicting the C_max_ of triptolide.** (A) The initial PLS loadings plot based on all the detected peaks/variables. Each point represents a metabolic feature detected in pre-dose serum using GC-MS. Red square, the selected variables of high VIP values were used to build the second PLS model for predicting the AUC (high dose). (B) The secondary PLS loadings plot based on the selected peaks with high VIP values. (C) and (D) represent first and second PLS models at low dose, respectively. Glu, glutamic acid; Cre, creatinine.
(TIF)

Figure S3 **The proposed fragmentation mechanism of triptolide GSH-conjugated metabolites.**
(TIF)

Figure S4 **Relative abundances of glutamic acid in serum and triptolide and its major metabolites in bile.** (A) The levels of glutamic acid in baseline serum; (B) the levels of triptolide in rat bile; (C) Relative LC/MS response intensity Metabolites of triptolide in bile. GSH, triptolide-GSH conjugate; O+GSH, GSH conjugate of the mono-hydroxylated triptolide. *significant difference vs. control group (p<0.05). #significant difference vs. CR group(p<0.05).
(TIF)

Figure S5 **the correlation between glutamic acid or creatinine and body weight.** (A) glutamic acid vs. body weight; (B) creatinine vs. body weight.
(TIF)

Table S1 **The metabolites of high VIP values in a two-stage PLS models correlating to PK parameters.**
(DOC)

Table S2 **The relative abundances of triptolide and its metabolites in bile.**
(DOC)

Table S3 **The equations and coefficients after linear regression between PK parameters and body weights.**
(DOC)

Table S4 **The equations and coefficients after linear regression between PK parameters and the normalized metabolites concentrations (against body weight).**
(DOC)
