# Supplementary figures and images for: Prediction of the Pharmacokinetic Parameters of Triptolide in Rats Based on Endogenous Molecules in Pre-Dose Baseline Serum
Source: PLoS One. 2012 Aug 17;7(8):e43389. doi: 10.1371/journal.pone.0043389 (PMC3422234; doi:10.1371/journal.pone.0043389)

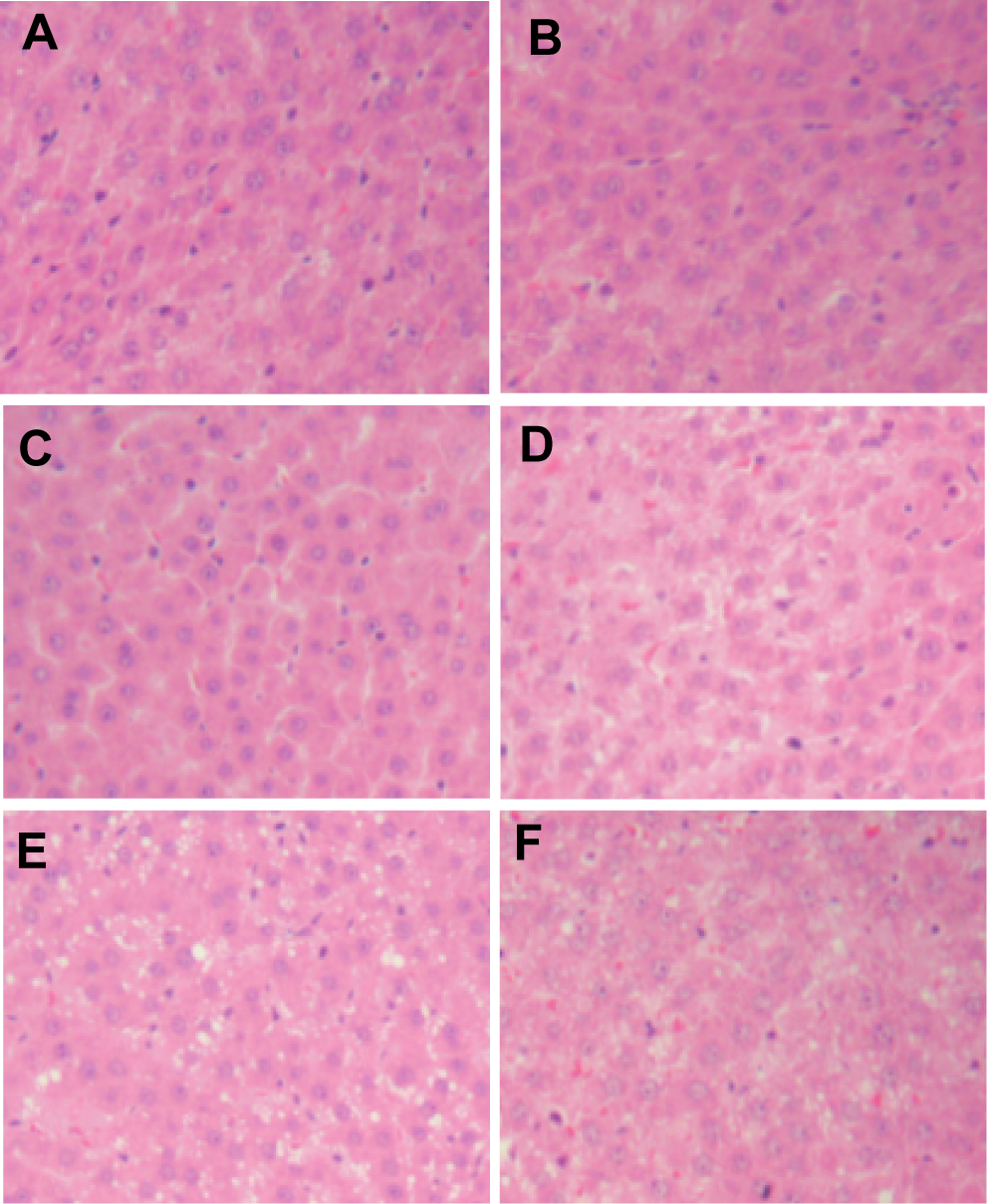

Supplement: Figure S1 — Histopathological inspection of the livers (HE staining) of rats with or without triptolide(0.6 mg/kg). A: Control+Non-treated; B: Control+ treated; C: CR+Non-treated; D: CR+ treated; E: HFD+Non-treated; F: HFD+ treated. (TIF) [file pone.0043389.s001.tif]

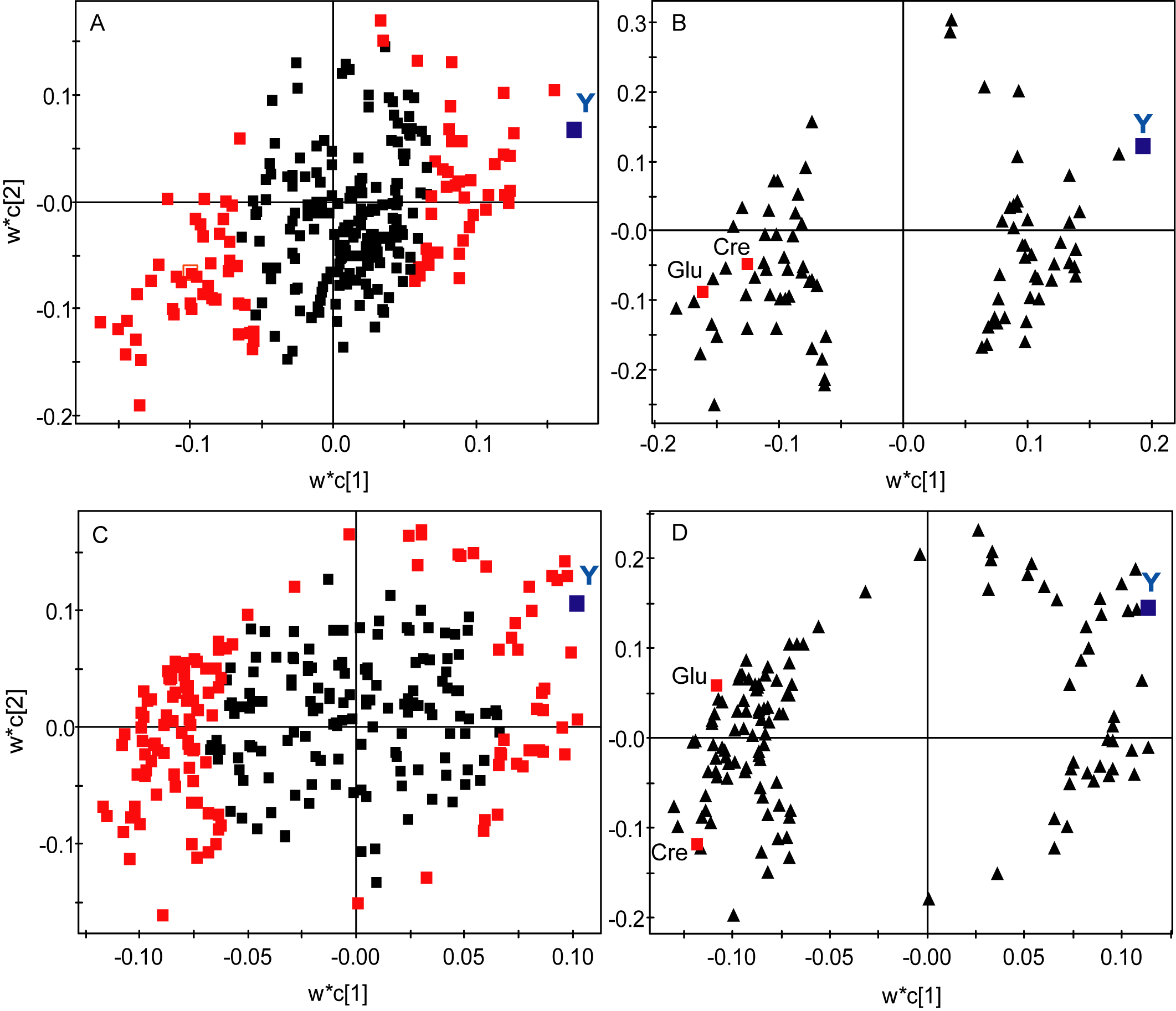

Supplement: Figure S2 — PLS loading plots for predicting the Cmax of triptolide. (A) The initial PLS loadings plot based on all the detected peaks/variables. Each point represents a metabolic feature detected in pre-dose serum using GC-MS. Red square, the selected variables of high VIP values were used to build the second PLS model for predicting the AUC (high dose). (B) The secondary PLS loadings plot based on the selected peaks with high VIP values. (C) and (D) represent first and second PLS models at low dose, respectively. Glu, glutamic acid; Cre, creatinine. (TIF) [file pone.0043389.s002.tif]

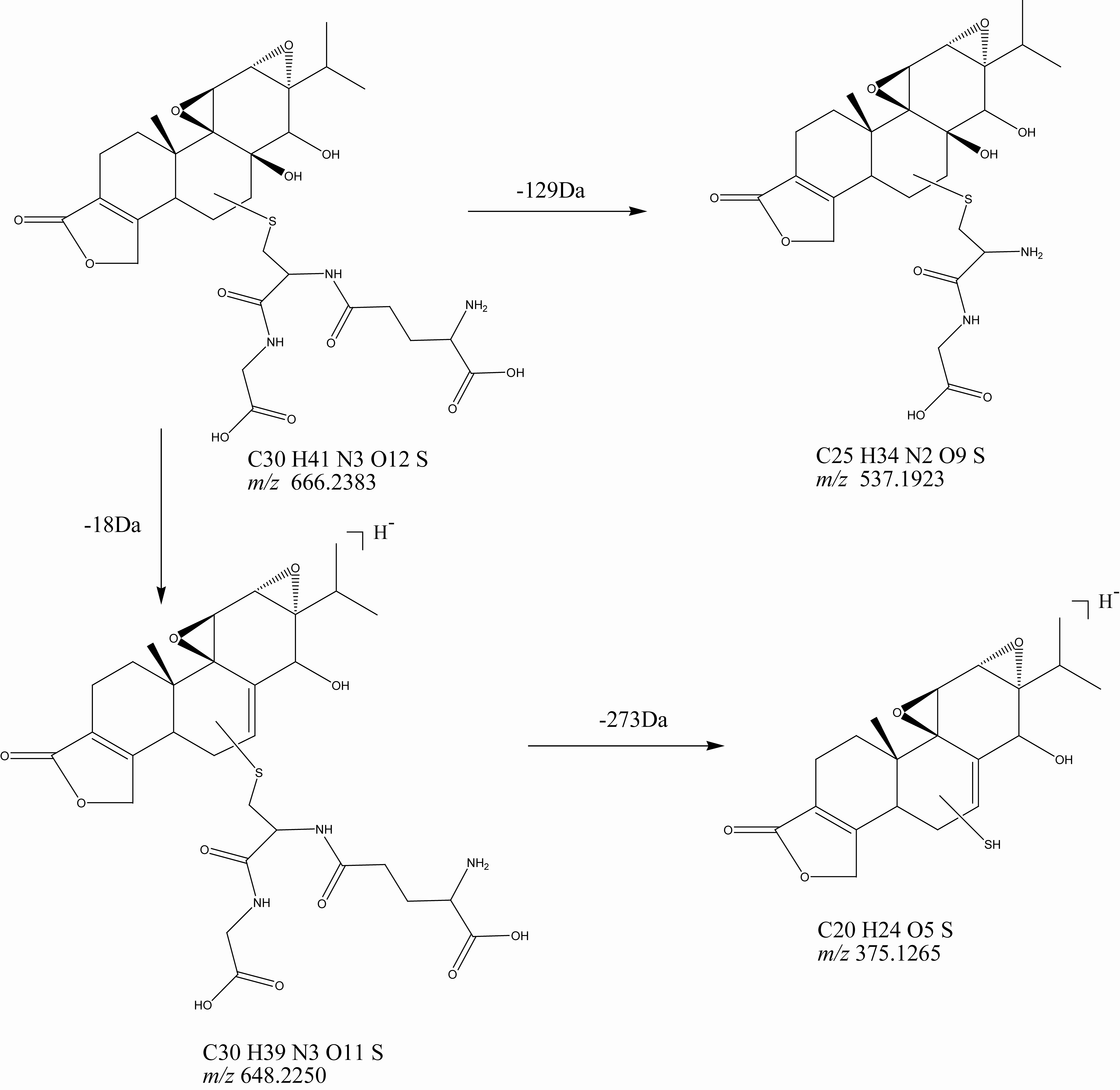

Supplement: Figure S3 — The proposed fragmentation mechanism of triptolide GSH-conjugated metabolites. (TIF) [file pone.0043389.s003.tif]

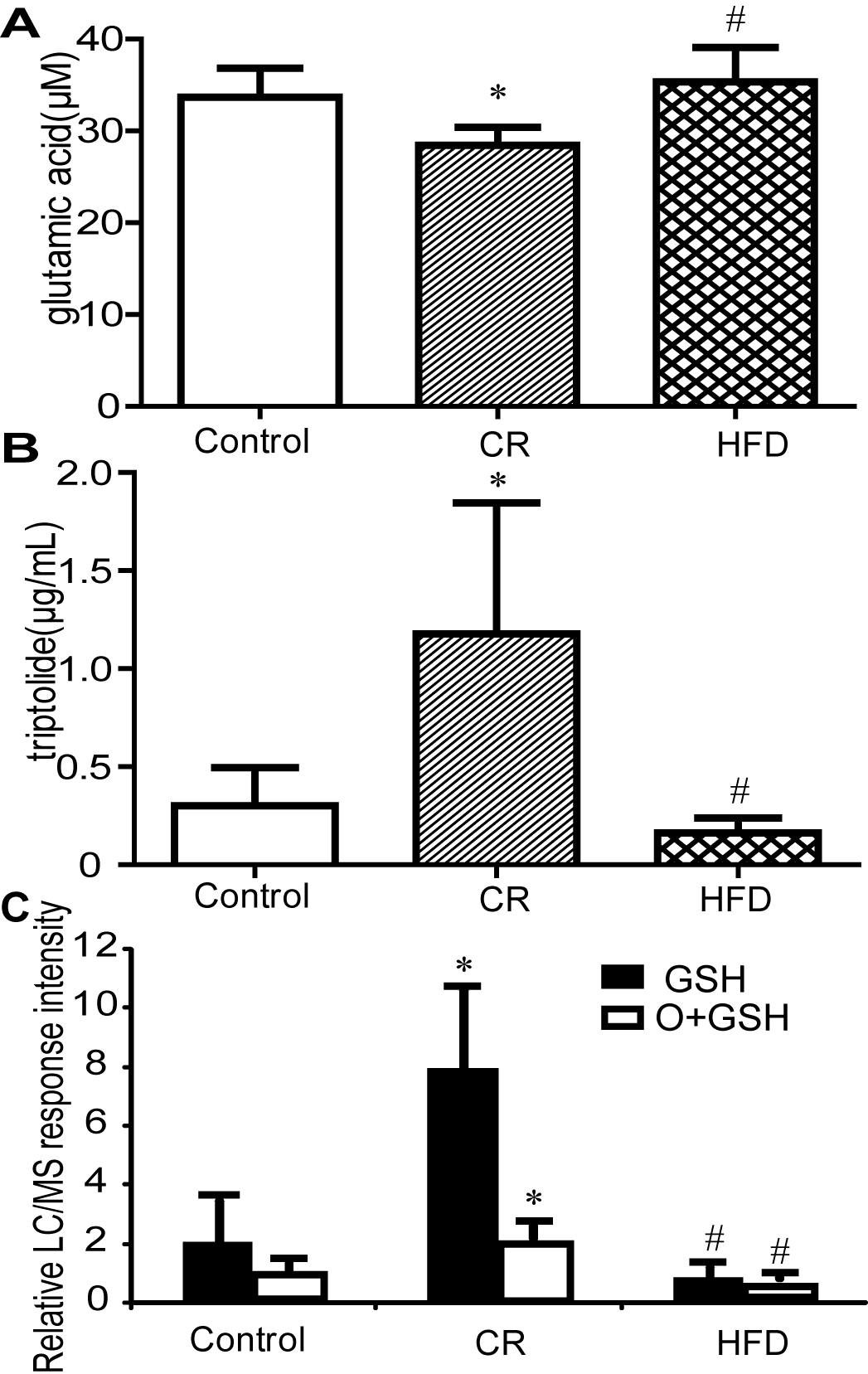

Supplement: Figure S4 — Relative abundances of glutamic acid in serum and triptolide and its major metabolites in bile. (A) The levels of glutamic acid in baseline serum; (B) the levels of triptolide in rat bile; (C) Relative LC/MS response intensity Metabolites of triptolide in bile. GSH, triptolide-GSH conjugate; O+GSH, GSH conjugate of the mono-hydroxylated triptolide. *significant difference vs. control group (p<0.05). #significant difference vs. CR group(p<0.05). (TIF) [file pone.0043389.s004.tif]

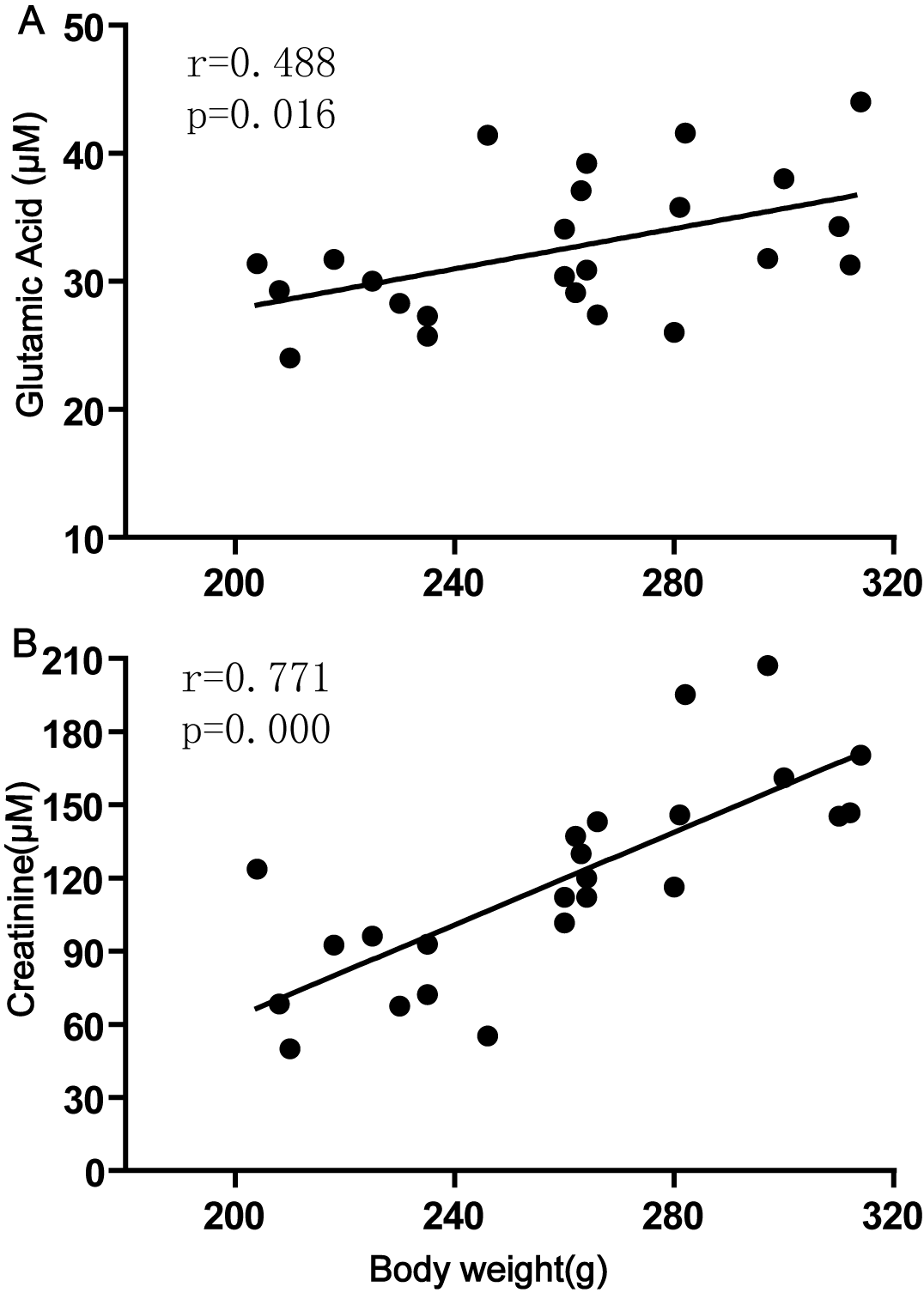

Supplement: Figure S5 — the correlation between glutamic acid or creatinine and body weight. (A) glutamic acid vs. body weight; (B) creatinine vs. body weight. (TIF) [file pone.0043389.s005.tif]
