# Supplementary material for: Prediction of the Pharmacokinetic Parameters of Triptolide in Rats Based on Endogenous Molecules in Pre-Dose Baseline Serum
Source: PLoS One. 2012 Aug 17;7(8):e43389. doi: 10.1371/journal.pone.0043389 (PMC3422234; doi:10.1371/journal.pone.0043389)
Supplement: Table S1 — The metabolites of high VIP values in a two-stage PLS models correlating to PK parameters. (DOC) [file pone.0043389.s006.doc]

Table S1. The metabolites of high VIP values in a two-stage PLS models correlating to PK parameters

| Parameter | Compounds | low dose | |  | high dose | |
| --- | --- | --- | --- | --- | --- | --- |
|  |  | 1^st^ step | 2^nd^ step |  | 1^st^ step | 2^nd^ step |
| Cmax | Glutamic acid | 1.31 | 1.09 |  | 2.21 | 1.40 |
|  | Creatinine | 1.56 | 1.25 |  | 1.67 | 1.07 |
|  | Valine | 1.32 | 0.99 |  | 1.74 | 1.11 |
|  | Lactic acid | 1.67 | 1.29 |  | 1.82 | 0.85 |
|  | Glyceric acid | 1.06 | 0.86 |  | 2.00 | 1.30 |
|  | Ornithine | 1.27 | 1.01 |  | 1.41 | 0.91 |
|  | Alpha-Ketoglutaric acid | 1.10 | 0.90 |  | 1.40 | 0.85 |
|  | Alpha-Tocopherol | 1.20 | 1.01 |  | 1.04 | 0.72 |
|  | 3-Hydroxy-Butanoic acid | 1.38 | 1.09 |  | 1.11 | 0.76 |
|  | 9-(Z)-Hexadecenoic acid | 1.70 | 1.31 |  | 0.21 | - |
|  | Uric acid | 1.24 | 1.06 |  | 1.22 | 1.56 |
|  | Octadecanoic acid | 1.33 | 1.10 |  | 0.85 | - |
|  | Lysine | 0.85 | - |  | 1.20 | 1.03 |
| AUC | Glutamic acid | 1.34 | 1.07 |  | 2.20 | 1.39 |
|  | Creatinine | 1.65 | 1.01 |  | 1.66 | 1.08 |
|  | Valine | 1.37 | 0.99 |  | 1.08 | 0.98 |
|  | Lactic acid | 1.67 | 0.82 |  | 1.51 | 0.72 |
|  | Glyceric acid | 1.13 | 1.13 |  | 1.84 | 1.21 |
|  | Ornithine | 1.38 | 1.10 |  | 1.32 | 0.87 |
|  | Alpha-Ketoglutaric acid | 1.20 | 0.86 |  | 1.30 | 0.78 |
|  | Glutamine | 0.91 | - |  | 1.70 | 1.49 |
|  | Lysine | 0.42 | - |  | 1.47 | 0.90 |
|  | Phenylalanine | 1.58 | 1.03 |  | 1.4 | 0.83 |
|  | Glucose | 1.48 | 1.10 |  | 1.32 | 0.87 |
|  | Isoleucine | 1.07 | 0.98 |  | 1.28 | 0.84 |
|  | Pyroglutamic acid | 0.64 | - |  | 1.21 | 0.90 |
|  | Docosahexaenoic acid | 0.83 | - |  | 1.4 | 0.96 |
|  | Xylitol | 1.26 | 1.02 |  | 1.45 | 0.78 |
|  | Hypoxanthine | 0.36 | - |  | 1.01 | 1.19 |
|  | Glycerol-3-phosphate | 1.15 | 1.19 |  | 0.95 | - |

- The VIP value <1 at 1^st^ step and exclude in 2^nd^ step.
