# Supplementary material for: Prediction of the Pharmacokinetic Parameters of Triptolide in Rats Based on Endogenous Molecules in Pre-Dose Baseline Serum
Source: PLoS One. 2012 Aug 17;7(8):e43389. doi: 10.1371/journal.pone.0043389 (PMC3422234; doi:10.1371/journal.pone.0043389)
Supplement: Table S2 — The relative abundances of triptolide and its metabolites in bile. (DOC) [file pone.0043389.s007.doc]

Table S2. The relative abundances of triptolide and its metabolites in bile

| Groups (n=6) | CR | Control | HFD |
| --- | --- | --- | --- |
| Triptolide in bile (ng/mL) | 1176.97 | 301.42 | 163.19 |
| Total amount of metabolites* | 8.03 | 19.35 | 25.20 |
| 1. Dihydroxylated triptolide (a/b) | 5.04/1.09 | 16.47/1.00 | 23.01/0.83 |
| 2. GSH conjugate (a/b) | 2.40/3.83 | 2.22/1.00 | 1.58/0.42 |
| 3. mono-Hydroxylated triptolide-GSH conjugate (a/b) | 0.58/3.14 | 0.66/1.00 | 0.61/0.55 |

CR, calorie restricted; HFD, high fat diet.

*: the sum of relative amount of metabolites 1, 2, 3.

a: The relative amount of metabolites normalized against triptolide in bile.

b: The relative amount of metabolites normalized against that of control group.
