# Supplementary material for: Prediction of the Pharmacokinetic Parameters of Triptolide in Rats Based on Endogenous Molecules in Pre-Dose Baseline Serum
Source: PLoS One. 2012 Aug 17;7(8):e43389. doi: 10.1371/journal.pone.0043389 (PMC3422234; doi:10.1371/journal.pone.0043389)
Supplement: Table S3 — The equations and coefficients after linear regression between PK parameters and body weights. (DOC) [file pone.0043389.s008.doc]

Table S3. The equations and coefficients after linear regression between PK parameters and body weights

| Y vector | X vector | Dose  (mg/kg) | Equation | Coefficients  and p values |
| --- | --- | --- | --- | --- |
| C_max_ | Body weight | 1.8 | C_max_=6.679-0.013weight(g) | r=0.374  p=0.232 |
| AUC | Body weight | 1.8 | AUC_0-30min_=10.073-0.017weight(g) | r=0.371  p=0.236 |
| C_max_ | Body weight | 0.6 | C_max_=10.218-0.031weight(g) | r=0.711  p=0.014 |
| AUC | Body weight | 0.6 | AUC_0-30min_ =10.859-0.024weight(g) | r=0.607  p=0.048 |
