# Supplementary material for: Prediction of the Pharmacokinetic Parameters of Triptolide in Rats Based on Endogenous Molecules in Pre-Dose Baseline Serum
Source: PLoS One. 2012 Aug 17;7(8):e43389. doi: 10.1371/journal.pone.0043389 (PMC3422234; doi:10.1371/journal.pone.0043389)
Supplement: Table S4 — The equations and coefficients after linear regression between PK parameters and the normalized metabolites concentrations (against body weight). (DOC) [file pone.0043389.s009.doc]

Table S4. The equations and coefficients after linear regression between PK parameters and the normalized metabolites concentrations(against body weight)

| Y vector | X vector | Dose  (mg/kg) | Equation | Coefficients  and p values |
| --- | --- | --- | --- | --- |
| C_max_ | creatinine glutamic acid | 1.8 | C_max_=6.239-7.333Glu-4.555Cre | r=0.362  p=0.531 |
| AUC | creatinine glutamic acid | 1.8 | AUC_0-30min_=9.88-10.849Glu-5.402Cre | r=0.363  p=0.530 |
| C_max_ | creatinine glutamic acid | 0.6 | C_max_=7.967-19.646Glu-7.991Cre | r=0.729  p=0.035 |
| AUC | creatinine glutamic acid | 0.6 | AUC_0-30min_=9.089-16.992Glu-5.162Cre | r=0.574  p=0.166 |
